# Supplementary material for: Electrospun Graphene Nanosheet-Filled Poly(trimethylene terephthalate) Composite Fibers: Effects of the Graphene Nanosheet Content on Morphologies, Electrical Conductivity, Crystallization Behavior, and Mechanical Properties
Source: Polymers (Basel). 2019 Jan 17;11(1):164. doi: 10.3390/polym11010164 (PMC6401836; doi:10.3390/polym11010164)
Supplement: Supplementary file 1 [file polymers-11-00164-s001.pdf]

Supplementary Material

Electrospun graphene nanosheet-filled poly(trimethylene terephthalate) composite fibers: effects of the graphene nanosheet content on morphologies, electrical conductivity, crystallization behavior, and mechanical properties

Chien-Lin Huang*[1], Hsuan-Hua Wu[1], Yung-Ching Jeng, and Wei-Zhi Liang [1]

[1]Department of Fiber and Composite Materials, Feng Chia University, Taichung 40724, Taiwan.

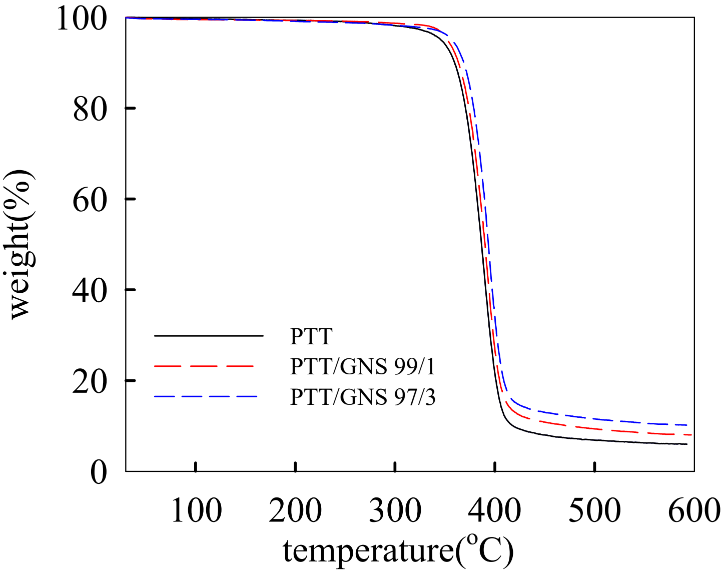

Figure S1. thermo-gravimetric analysis curves of the PTT/GNS CFMs.

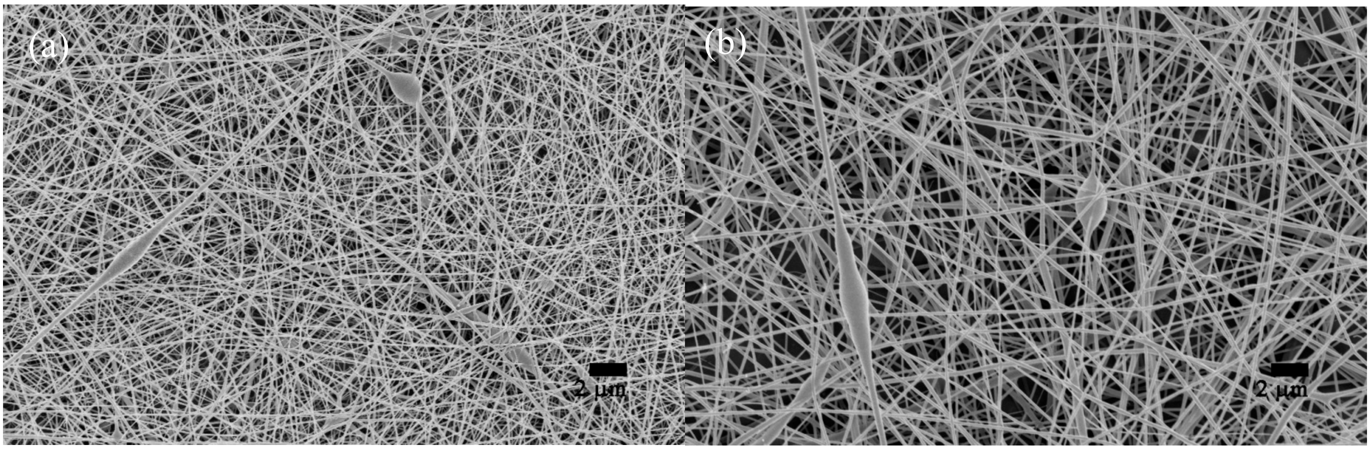

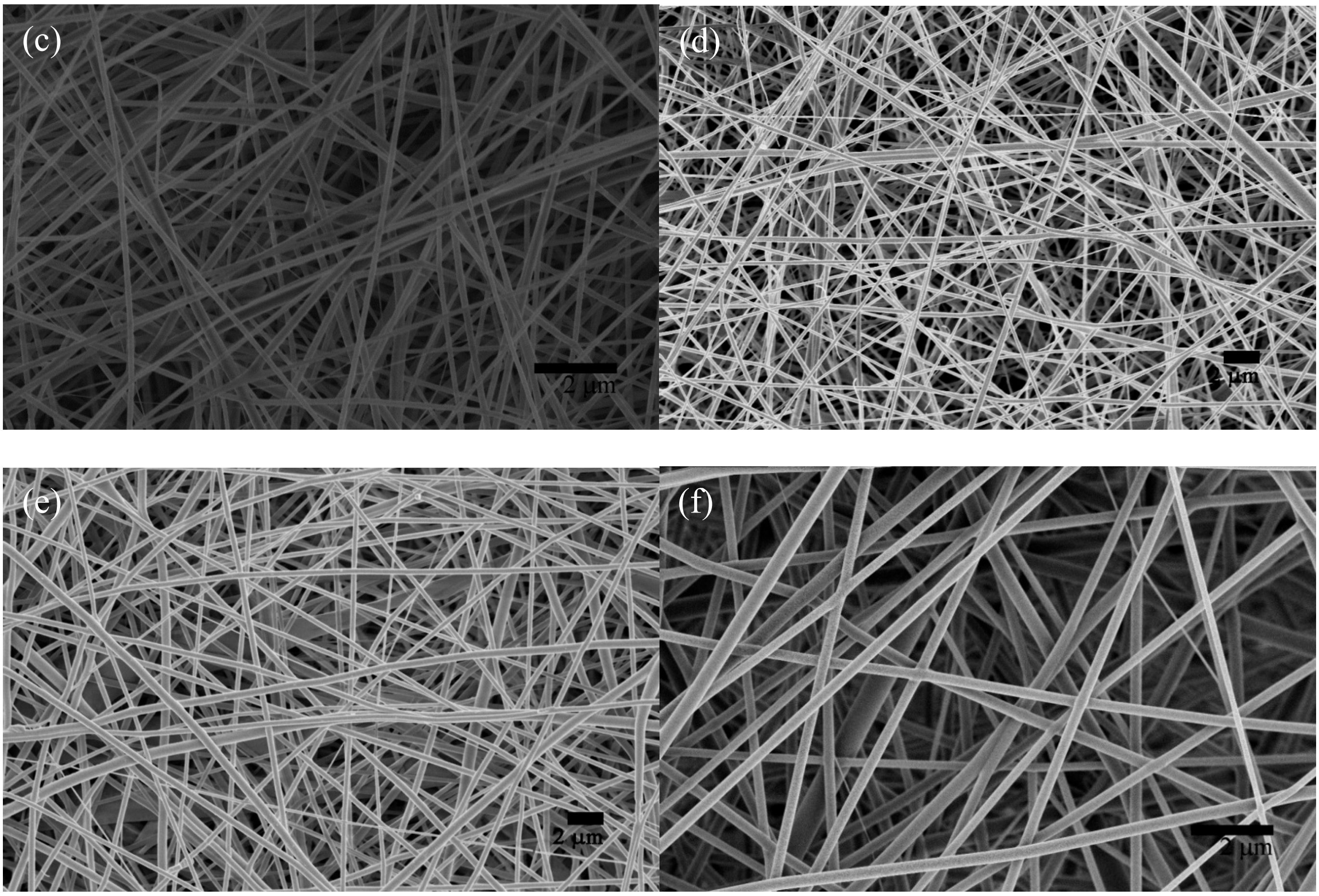

Figure S2. SEM images of electrospun fibers from (a) 7 wt% PTT/TFA solution, (b) 8 wt% PTT/TFA solution, (c) 9 wt% PTT/TFA solution, (d) 11 wt% PTT/TFA solution, (e) 12wt% PTT/TFA solution, and (f) 14 wt% PTT/TFA solution.

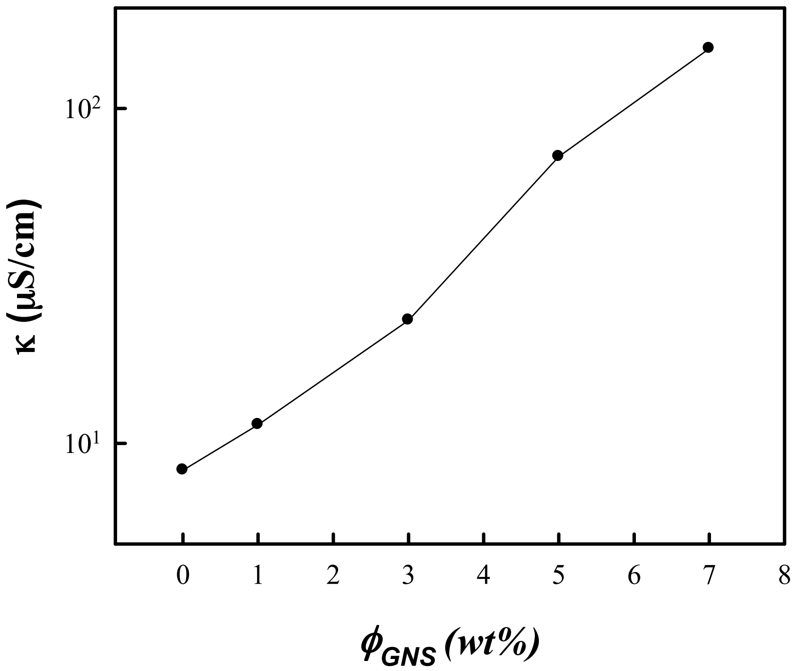

Figure S3. Effects of GNS concentration on PTT solution conductivity at 25 ºC.

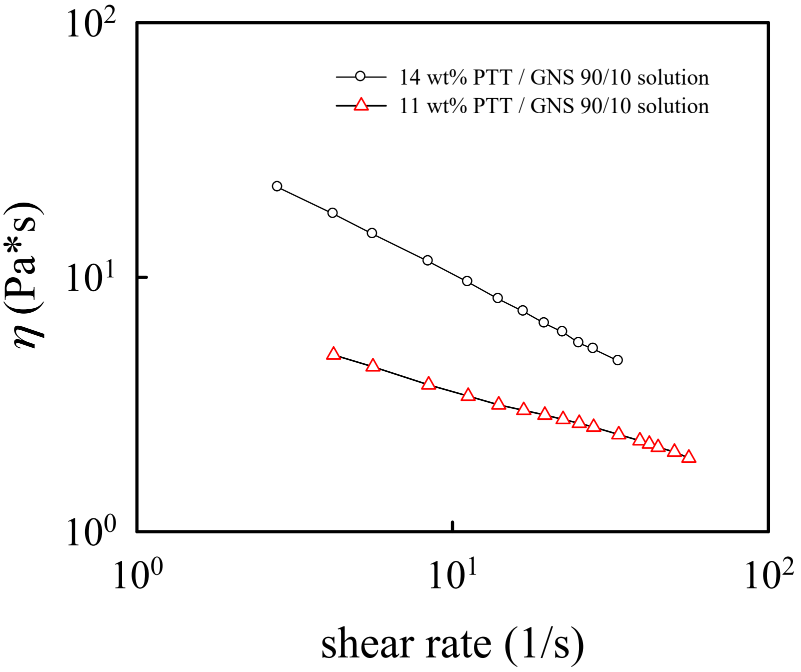

Figure S4. Effects of PTT/TFA concentration with 10 wt% GNS content on solution viscosity.

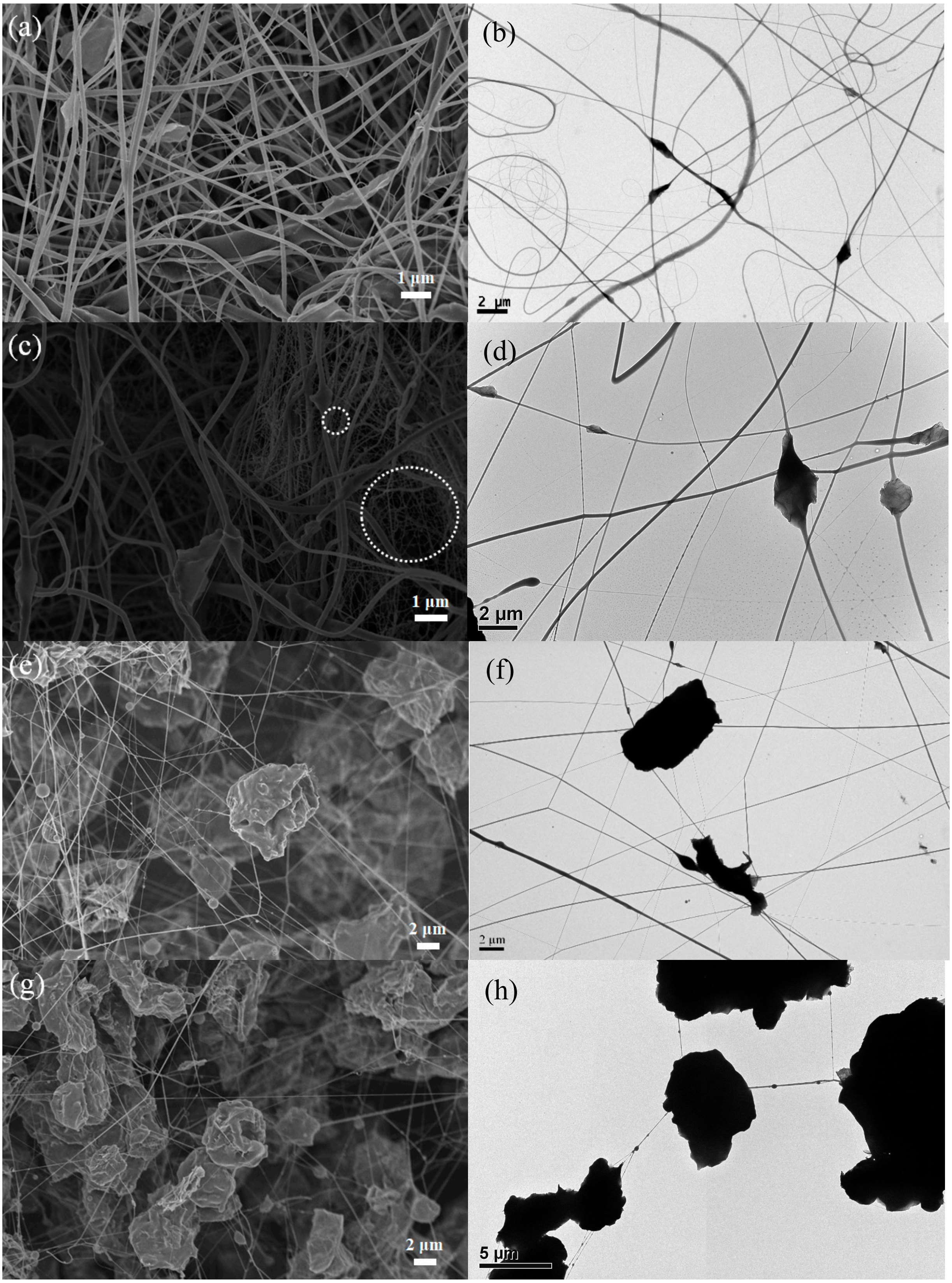

Figure S5. SEM and TEM images of electrospun PTT fibers filled with: (a), (b) 10 wt%, (c), (d) 12 wt%, (e), (f) 14 wt%, and (g), (h) 16 wt% GNS.

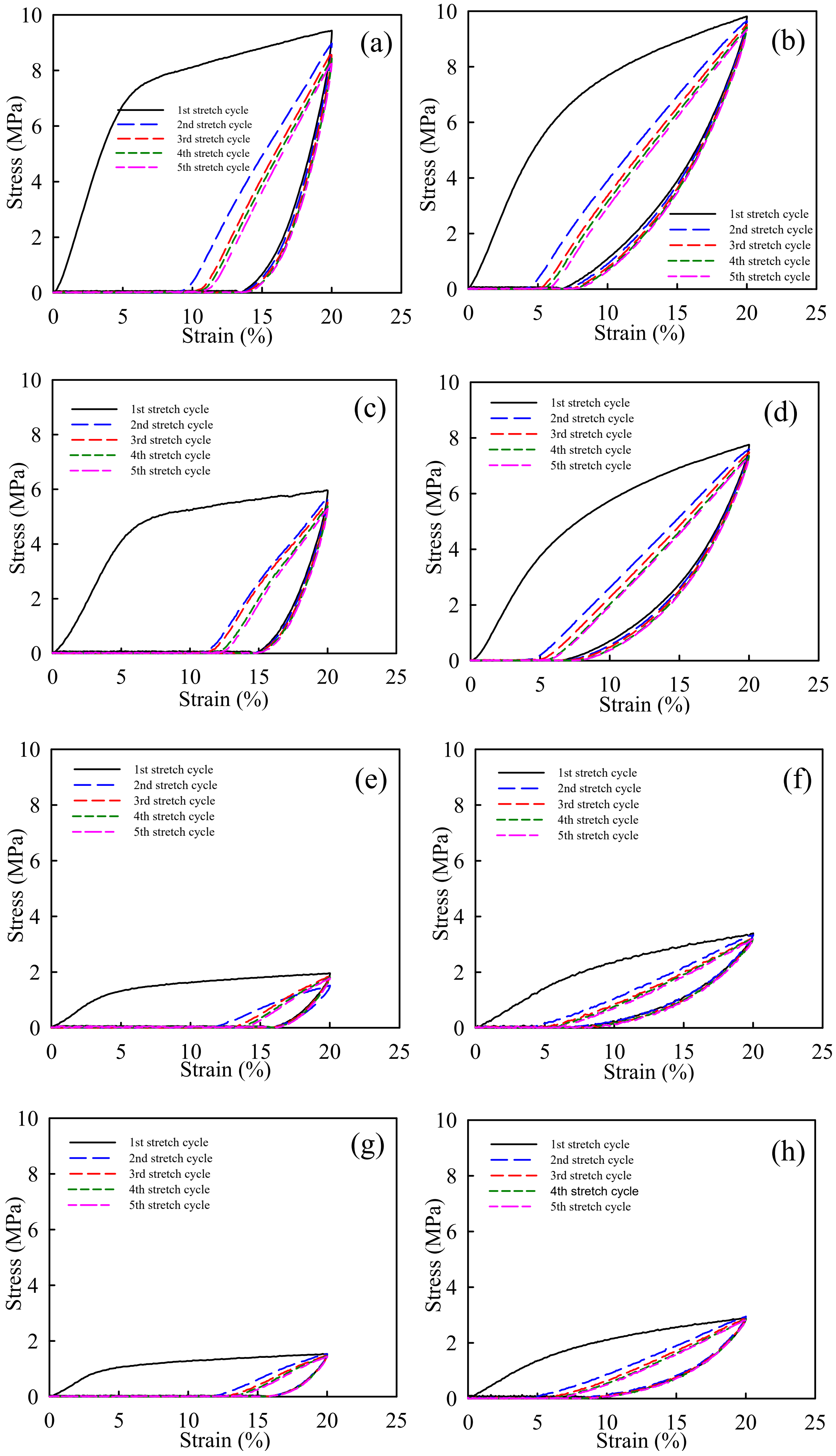

Figure S6. Elastic recovery of the (a) neat PTT CFMs, (b) neat PTT CFMs with annealing at 170 ºC, (c) PTT/GNS 99/1 CFMs, (d) PTT/GNS 99/1 CFMs with annealing at 170 ºC, (e) PTT/GNS 97/3 CFMs, (f) PTT/GNS 97/3 CFMs with annealing at 170 ºC, (g) PTT/GNS 95/5 CFMs, and (b) PTT/GNS 95/5 CFMs with annealing at 170 ºC during five cycles of loading–unloading with an elongation of 20%.
